# Recombinant cellular model system for human muscle-type nicotinic acetylcholine receptor α1_2_β1δε

**DOI:** 10.1007/s12192-023-01395-0

**Published:** 2023-11-25

**Authors:** Sabrina Brockmöller, Thomas Seeger, Franz Worek, Simone Rothmiller

**Affiliations:** Bundeswehr Institute of Pharmacology and Toxicology, Munich, Germany

**Keywords:** Nicotinic acetylcholine receptor, Molecular chaperones, Nicotine

## Abstract

The human muscle-type nicotinic acetylcholine receptor α1_2_β1δε (nAChR) is a complex transmembrane receptor needed for drug screening for disorders like congenital myasthenic syndromes and multiple pterygium syndrome. Until today, most models are still using the nAChR from *Torpedo californica* electric ray. A simple reproducible cellular system expressing functional human muscle-type nAChR is still missing. This study addressed this issue and further tested the hypothesis that different chaperones, both biological and chemical, and posttranslational modification supporting substances as well as hypothermic incubation are able to increase the nAChR yield. Therefore, Gibson cloning was used to generate transfer plasmids carrying the sequence of nAChR or chosen biological chaperones to support the nAChR folding in the cellular host. Viral transduction was used for stable integration of these transgenes in Chinese hamster ovary cells (CHO). Proteins were detected with Western blot, in-cell and on-cell Western, and the function of the receptor with voltage clamp analysis. We show that the internalization of nAChR into plasma membranes was sufficient for detection and function. Additional transgenic overexpression of biological chaperones did result in a reduced nAChR expression. Chemical chaperones, posttranslational modification supporting substances, and hypothermic conditions are well-suited supporting applications to increase the protein levels of different subunits. This study presents a stable and functional cell line that expresses human muscle-type nAChR and yields can be further increased using the chemical chaperone nicotine without affecting cell viability. The simplified access to this model system should enable numerous applications beyond drug development.

**Graphical abstract:**

**Figure:**
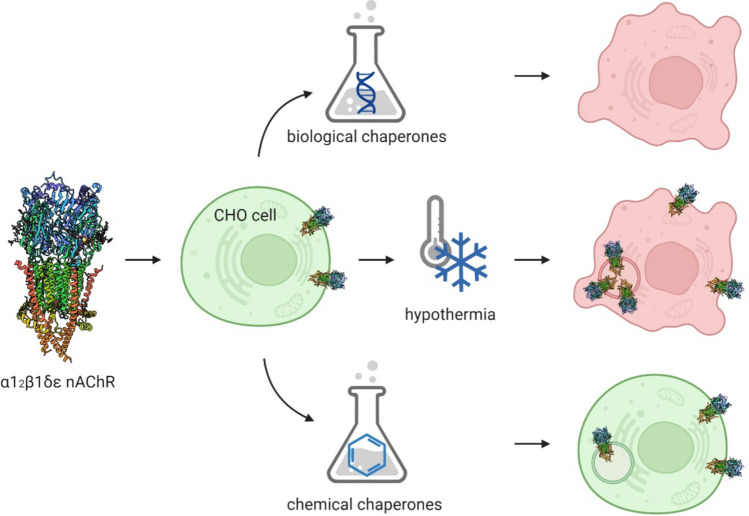
Graphical abstract created with http://biorender.com

**Supplementary Information:**

The online version contains supplementary material available at 10.1007/s12192-023-01395-0.

## Introduction

Nicotinic acetylcholine receptors (nAChR) exist in neuronal and muscle subtypes (Millar and Gotti 2009), and neurotransmission is their key role in brain or muscular junctions. Acetylcholine binds to the receptor in the postsynaptic cleft, the receptor changes its conformation, and cholinergic signal transduction is induced (Wessler and Kirkpatrick 2008). The nAChR is a pentamer transmembrane protein and every subunit permeates the plasma membrane four times (Unwin 2005). The composition of the five subunits defines the subtype of nAChR, and the human adult muscle-type nAChR consists of the subunits α1_2_β1δε (Millar and Gotti 2009). Until today, no functional stable expression model system of the human adult muscle-type α1_2_β1δε exists. The establishment of the human muscle-type nAChR as a cellular model system would allow the development of drug screening assays for disorders like congenital myasthenic syndromes and multiple pterygium syndrome.

For decades, biotechnology methods were used to create cell lines to produce recombinant target proteins. In the case of the muscle nAChR, such a cellular system is desired. Until now, there are some model systems based on *Xenopus* expression (Mishina et al. 1986; Jonsson Fagerlund et al. 2009), animal species muscle type in some cell lines, or chimeras in muscle cells (Forsayeth et al. 1990; Gu et al. 1990; Green and Claudio 1993; Rudell et al. 2014). The most widely used model today is the muscle nAChR of the *Torpedo californica* electric ray. The disadvantage of this model system is that results are generated from a receptor from a non-human species. To maximize the transferability of *in vitro* results for drug research, the possible drug candidates should be tested on a human receptor model. A major issue for this recombinant cellular model is entering the transgene information of four different subunits stably into the genome. After stable transfection, the main challenges of recombinant nAChR expression are correct folding, assembly, posttranslational modifications, insertion into the plasma membrane, and neurotransmitter function.

The aim of this study was to establish a cellular model for the human α1_2_β1δε nAChR and whether the receptor’s yields can be increased by (i) co-expression of biological chaperones like CN, BiP, and rapsyn, (ii) chemical chaperones, (iii) substances which support posttranslational modification, or (iv) hypothermic incubation.

Biological chaperones support the correct folding of the receptor which prevents degradation by endoplasmic reticulum–associated degradation (ERAD) because of assembly errors. For this study, only chaperones that were previously shown to specifically support the muscle-type nAChR were selected. Calnexin (CN) associates with single subunits and prevents nAChR from degradation after tagging with ubiquitin, which would usually result in degradation by the 26S proteasome (Keller and Taylor 1999). Binding immunoglobulin protein (BiP) supports homeostasis in ER (Lièvremont et al. 1997) and associates only with nascent subunits of nAChR (Blount and Merlie 1991; Forsayeth et al. 1992). BiP also interacts with ERAD and thus controls translation modification in the ER (Otero et al. 2010). The shuttle protein receptor-associated protein at the synapse (rapsyn) transports the nAChR from the trans-Golgi network to the plasma membrane (Marchand et al. 2000; Marchand et al. 2002), and induced clustering and internalization of nAChR into the plasma membrane (Fuhrer and Huh 2002).

Besides biological chaperones, chemical chaperones may also play a role as nicotine induces neuronal nAChR folding (Henderson and Lester 2015). In this study, nicotine, choline, cysteine, and tetramethylammonium (TMA) were tested whether they support the assembly and thus result in increased receptor yields. Substances supporting the posttranslational modification are forskolin, lactacystin, MG-132, and suberoylanilide hydroxamic acid (SAHA).

Hypothermia was also tested whether it could increase nAChR yields in our study since positive effects of hypothermia could be shown for neuronal nAChR (Nelson et al. 2001). Under hypothermia, cell proliferation is decreased by reduced metabolism (Fox et al. 2004; Yoon et al. 2005); and under cold stress conditions, more stable mRNAs are synthesized (Yoon et al. 2003b; Yoon et al. 2003a).

Receptor expression and internalization were detected by Western blot, in-cell Western, and on-cell Western, and functional analysis of nAChR was performed by voltage clamp. Receptor yields could be increased by the application of chemical chaperones or hypothermia, but hypothermia simultaneously reduced cell viability. Here, we present an established stable and functional Chinese hamster ovary (CHO) cell line expressing recombinant human muscle-type nAChR.

## Material and methods

### Cloning strategy

*Stbl3* (C737303 Invitrogen) were transformed with 5-ng cloning vectors. These vectors included synthesized sequences of human muscle nAChR (Uniprot data P02708 α1, P11230 β1, Q07001 δ, and Q04844 ε) and of chaperones (Uniprot data Q13702 rapsyn, P27824 calnexin, and P11021 BiP), which were obtained from Geneart Regensburg. The insert sequence of nAChR consisted of a splenic focus forming virus (SFFV) promoter between β1- and δ subunits and puromycin resistance. Every subunit was tagged: α1 His-tag, β1 hemagglutinin (HA)-tag, δ myc-tag, and ε flag-tag. Insert sequence of the chaperones consisted of rapsyn and an SFFV promoter upstream calnexin and BiP as well as a blasticidin resistance. Every chaperone was tagged: rapsyn strep-tag II, calnexin rho1D4-tag, and BiP Ty1-tag. Plasmid purification was performed with Spin Miniprep Kit by QIAGEN. Plasmids were digested with *AgeI-HF* (R355L) and *SpeI-HF* (R3133L) from New England Biolabs. Inserts were purified with ReliaPrep DNA clean-up and concentration system by Promega, after agarose gel electrophoresis in E-Gel Power Snap by Invitrogen with E-Gel EX 2% agarose gels (G401002 Invitrogen). For elution, 37 °C RNase-free water was used which incubates on the column for 4 min at 37 °C.

**Fig. 1:**
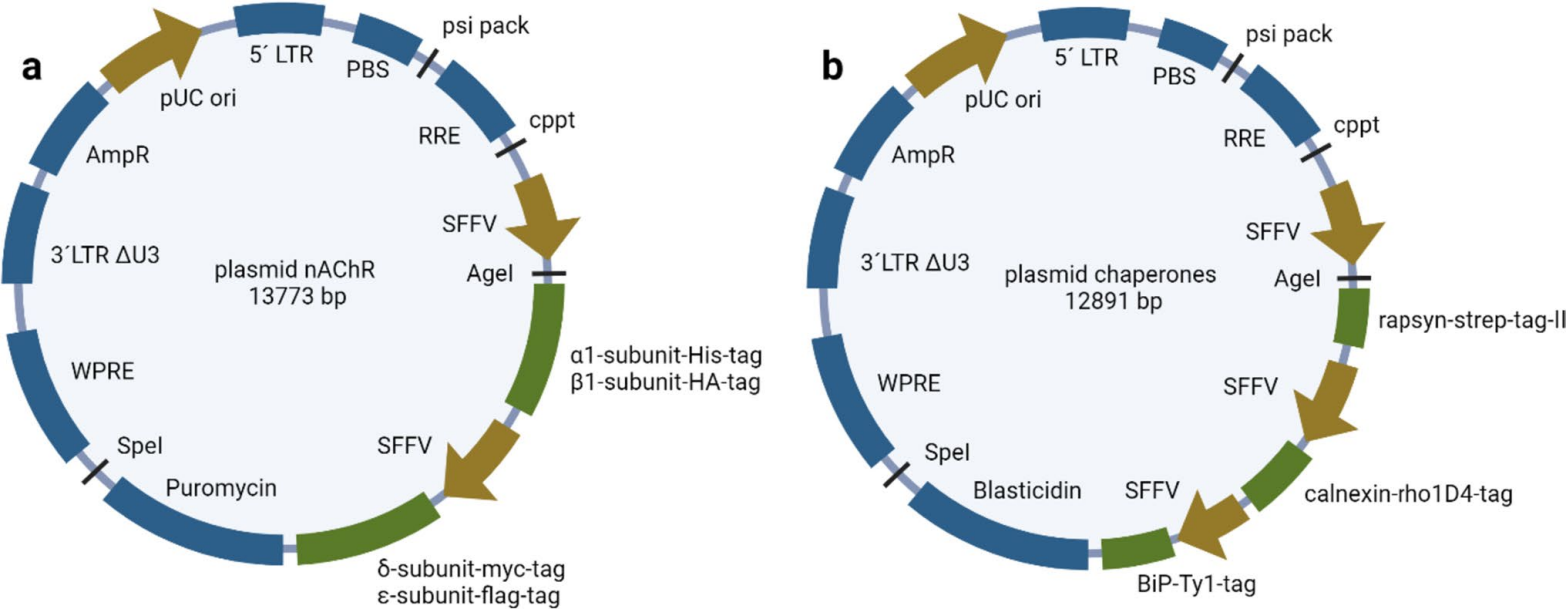
Plasmid maps of both transfer plasmids. Vector backbone is the construct of pL-SFFV.Reporter.RFP657.PAC (61395 Addgene) which was cloned via restriction sites *AgeI* and *SpeI*. **a** Insert for nAChR consists of a sequence of α1 subunit with His-tag, β1 subunit with HA-tag, SFFV promotor, δ subunit with myc-tag, ε subunit with flag-tag, and puromycin antibiotic resistance. **b** Insert for chaperones consists of a sequence of rapsyn with strep-tag-II, SFFV promotor, calnexin with rho1D4-tag, SFFV promotor, BiP with Ty1-tag, and blasticidin antibiotic resistance. Abbreviations of plasmid maps: 5′LTR 5′long terminal repeat, PBS primer binding site, RRE reverse response element, cppt central polypurine tract, SFFV splenic focus forming virus promotor, WPRE Woodchuck hepatitis virus posttranscriptional regulatory element, 3′LTR ∆U3 3′long terminal repeat deleted, AmpR ampicillin antibiotic resistance, pUC ori origin of replication (created with http://biorender.com)

Gibson cloning was used to generate transfer plasmids. Empty backbone vector (61395 pL-SFFV.Reporter.RFP657.PAC-Emptyvector by Addgene) was digested with *AgeI-HF* and *SpeI-HF* and purified like the inserts described above. Cloning was performed with a 75-ng backbone vector in a ratio of 1:1 with a target insert. NEBuilder HiFi DNA Assembly Mastermix (E2621L New England Biolabs) was used with incubation at 50 °C for 30 min in a 30-μL approach. Fifteen nanograms was used for the transformation of *Stbl3*. Target plasmid purification was made with the NucleoBond Xtra Maxi Plus Kit (Macherey and Nagel). Purified target/transfer plasmids are shown in Fig. 1. Target plasmid correctness was verified by sequence analysis with Mix2Seq Kit (Eurofins Genomics).

### Cell culture

CHO and HEK293T cells were purchased from Leibniz Institute DSMZ (ACC 110, ACC 635). CHO cells were incubated in F-12 Nut Mix + GlutaMax^TM^ (31765-027 Gibco) with 9% fetal calf serum (FCS) (10270-106 Gibco) by 37 °C, 5% CO_2_, and 90% humidity. They were passaged at a confluence of 70–80%. PBS pH 7.4 (10010-015 Gibco) was used to wash and TrypLE^TM^ Express (12605-010 Gibco) to detach the cells. HEK293T cells were incubated under the same conditions with DMEM/F-12 (31331-028 Gibco) with 9% FCS. After transduction, incubation of CHO cells overexpressing nAChR was performed with 16.5% FCS in the medium.

### Viral transduction

For the third-generation lentiviral vector system, the transfer plasmid, two packaging plasmids (pMDLg_pRRE 12251 Addgene, pRSV-Rev 12253 Addgene), and the envelope plasmid (pMD2.G 12259 Addgene) were needed. To generate replication-incompetent lentivirus particles, HEK293T cells were used as a packaging system. The principle of packaging into virus particles was analogous to Seiler et al. (2008) and Ingold et al. (2018). In total, 0.5 × 10^6^ HEK296T cells were seeded per well in six-well plates. At a confluence of 50%, they were transfected in a ratio of five transfer plasmids: 2 pMDLg_pRRE: 10 pRSV-Rev: 5 envelope plasmid in a total of 2 μg DNA. The plasmids were complexed in a ratio of 1:3 with X-tremeGene HP DNA Transfection Reagent (06366236001 Sigma-Aldrich) and incubated for 15 min at room temperature in a FCS-free medium. A complex plasmid reagent was carefully pipetted to the cells. After 72 h, the supernatant of HEK293T cells was harvested and filtered with a 0.45-μm PVDF membrane syringe filter. Virus supernatant was used for one and two target transductions. One target transduction (1-TD) included one of the following transfer plasmids: (i) empty vector backbone with reporter RFP, (ii) vector backbone with chaperones, or (iii) vector backbone with nAChR only. In total, 25,000 CHO cells were seeded per well in six-well plates in a ratio of 1:1 of culture medium and one of the one-target virus particle supernatants. As support, the transduction medium contained 10 μg/mL protamine sulfate. After 48 h incubation, cells were selected every day with fresh 2 μg/mL puromycin or 0.5 μg/mL blasticidin in a culture medium. They were detached every second day. Two target transductions (2-TD) were both target vectors with nAChR and chaperones. Therefore, 12,500 CHO cells were seeded in six-well plates in a ratio of 1:1:1 culture medium and both virus particle supernatants. This transduction was supported by 20 μg/mL protamine sulfate. After 48 h, the selection was obtained with both 2 μg/mL puromycin and 0.5 μg/mL blasticidin in culture medium. Cells were detached every 3 days. Further, the 2-TD was performed in direct transduction and in steps: two one-target transductions right after each other.

### Viability assay

All created stable transduced CHO cell lines were tested for their viability with Cell Proliferation Kit II (XTT Roche). Cells were seeded with 30,000 cells/well in 96-well plates in medium with 9% FCS or 16.5% FCS. After 24 h of incubation, the medium was changed to a ratio of 2:1 with XTT reagent. The absorbance of metabolic formation of formazan was measured by 450 nm and a reference by 630 nm wavelength in a microplate reader (TECAN infinite M200 PRO and i-control 1.8 SP1 Software). Data were normalized to the values of non-transduced cells in medium with 9% and 16.5% FCS.

### Western blot

For the verification of stable nAChR expression with Western blot analysis, at least 200 × 10^6^ cells were needed. Cells were incubated in T175 culture flasks and washed with PBS before they were detached. The cell suspension was centrifuged at 58×*g* for 5 min. The supernatant was discarded and the pellet was resuspended with 2 mL PBS. All cell suspensions were collected in a 50 mL Falcon and centrifuged at 58×*g* for 5 min again. After discarding the supernatant, the pellet was weighed and resuspended in a ratio of 1:10 in a solubilization buffer (2% sodium cholate in PBS with cOmplete Protease Inhibitor Cocktail (Roche)). Cell disruption was obtained by 60× dauncing in a 4 °C cold metallic manual dauncer. Cell suspension was incubated at 4 °C for 1 h. After that, the suspension was centrifuged at 700×*g* by 4 °C for 3 min to remove large cell debris. Afterwards, the supernatant was centrifuged again at 21,000×*g* by 4 °C for 1 h.

The solubilized nAChR supernatant was purified with Dynabeads His-tag isolation Kit (10104D Invitrogen). One hundred microliters of Ni-NTA coated magnetic beads was incubated with 200 μL solubilized nAChR suspension and 700 μL binding buffer (50 mM sodium phosphate pH 8.0, 300 mM sodium chloride, and 0.02% Tween 20) for 2 h at 800 rpm and 16 °C in a heating block. After that, beads that bind the His-tag from the α1 subunit of the nAChR were washed four times with 600 μL binding buffer. In every wash step, the DynaMag2 by Invitrogen was used. The nAChR was eluted with 200 μL elution buffer (350 mM imidazole, 50 mM sodium phosphate pH 8.0, 300 mM sodium chloride, and 0.001% Tween 20) by an incubation of 30 min, 800 rpm, and 16 °C in heating block. Afterward, eluted nAChR were separated from bead suspension with DynaMag2.

Per well within the SDS gel, 100–200 ng of the purified nAChR solution was applied. Samples were denatured with loading buffer (2% SDS, 0.5 M DTT, 5 μL 4X loading dye by LiCor and 8 M Urea) by incubation for 10 min at 60 °C. Sodium dodecyl sulfate polyacrylamide gel electrophoresis (SDS PAGE) was done with a 4–12% Bis-Tris gel (Invitrogen). The SDS PAGE runs on ice at 200 volts (V) for 50 min and MES SDS running buffer (NP0002) by Invitrogen. Three microliters of Chameleon Duo marker (928-60000 LiCor) was used. Blotting the separated proteins on the Immobilon FL membrane (IPL20200 Sigma-Aldrich) was performed with the wet blotting principle. Briefly, the membrane was activated in methanol. For the transfer of proteins, a transfer buffer from Invitrogen (NP00061 Invitrogen) was used. Blotting was performed for 1 h by 20 V on ice. Afterwards, the membrane was blocked for 1 h at room temperature in PBS blocking buffer (LiCor). Every subunit was detected separately. Antibodies against subunits were obtained from Abcam for denatured detection (α1 ab28489, β1 ab236959, δ ab233758, ε ab233831) and used 1:1000 diluted in blocking buffer with 0.2% Tween 20. Membranes were incubated overnight at 4 °C. After that, membranes were washed three times with PBST (0.1% Tween-20) for 5 min. The detection antibodies (IRDye 800 Mouse 2632210, IRDye 800 Rabbit 92632211 LiCor) were used diluted 1:10,000 in blocking buffer with 0.01% SDS and 0.2% Tween 20. Incubation was performed for 1 h at room temperature and protected from light. Membranes were washed three times with PBST for 5 min. Membranes were scanned with Odyssey CLx (LiCor) and images were analyzed with Image Studio software version 5.2 (LiCor).

### Electrophysiology

Cells were harvested and recorded according to the manufacturer’s standard procedures (Obergrussberger et al. 2014). In brief, CHO cells, which were chosen as cellular hosts since they are suitable for voltage clamp analysis (Scheffel et al. 2018b; Scheffel et al. 2018a), were detached as described in the section “Cell culture” and washed with 2 mM EDTA in PBS pH 7.4. Electrophysiological whole-cell recordings were conducted using Nanion’s Patchliner (Patchliner Octo, Nanion Technologies), an automated patch-clamp system with eight amplifier channels due to two HEKA EPC10 Quadro Amplifiers. All currents were elicited from single cells using a medium resistance NPC-16 borosilicate chip 1.8–3 MΩ (071102 by Nanion Technologies) at a holding potential of −70 mV. Buffer solutions external solution 083001, internal solution 083007, and seal solution 083012 (all by Nanion Technologies) were used. A fresh stock solution of 10 mM nicotine was used for each analysis. For receptor activation, 15 μL of 70 μM nicotine was applied for 132 ms at a rate of 114 μL/s, directly followed by 200 μL of external buffer solution for washout. The sampling rate was set to 50 kHz and filtered at 2.9 kHz.

### In-cell Western and on-cell Western

In the case of in-cell Western (ICW), 30,000 cells/well were seeded in black 96-well microplates with clear bottoms. After 24 h, cells were fixed in wells and permeabilized with −20 °C ice-cold 100% methanol for 10 min at room temperature. Then, methanol was removed and plates were washed three times with 150 μL/well PBS for 5 min. Cells were blocked with a blocking buffer (LiCor) for 1.5 h at 180 rpm at room temperature. After removing the blocking buffer, cells were incubated with primary antibody diluted 1:200 in blocking buffer for 2.5 h at 180 rpm at room temperature (Abcam antibodies for native detection: His-tag ab18184, HA-tag ab49969, myc-tag ab32, falg-tag ab236777, strep-tag-II ab180957, rho1D4-tag ab5417, RFP ab185921, intrinsic CN ab133615, intrinsic BiP ab213258, and Ty1-tag MA523513 by Thermo Fisher). After incubation, plates were washed three times for 5 min with PBST (0.1% Tween 20). Detection antibody diluted 1:800 in blocking buffer was incubated for 1 h protected from light and 180 rpm (IRDye 800 Mouse 2632210, IRDye 800 Rabbit 92632211, IRDye 680 Mouse 92668070, IRDye 680 Rabbit 92668071 LiCor). Finally, cells were washed three times for 5 min with PBST. Every well was filled with 100 μL PBS and plates were scanned with Odyssey CLx (LiCor) at 800 and 680 nm. Scans were analyzed with Image Studio software version 5.2 (LiCor). Emission signals were normalized to cell counts with Cell Tag 700 Stain (926-41090 LiCor). GAPDH (ab8245, Abcam) was used as positive control. On-cell Western (OCW) was almost the same procedure. The difference was in the permeabilization step, where cells were only fixed with 4% paraformaldehyde in PBS for 20 min at 4 °C. Integrin A5 (ab150361, Abcam) was used as a positive control.

### Genomic PCR and gel electrophoresis

Genomic PCR showed stable genome integration. Incubated cells were washed and detached as described above in the section “Cell culture” and centrifuged at 58×*g* for 5 min. The supernatant was discarded and the pellet was resuspended and lysed in DNAzol (10503027 Invitrogen) in a ratio of 20 × 10^6^ cells to 1 mL DNAzol. Precipitation of DNA was performed with 100% ethanol in double the volume of used DNAzol. DNA was washed twice with 1 mL of 75% ethanol and dried. DNA was solubilized in an 8 mM NaOH solution. PCR was performed with 50 ng/μL DNA concentration. Amplification of DNA fragments for every nAChR subunit was performed with the PCR protocol shown in Table 1. In Table 2, the primers and DMSO concentrations used for the different fragments are listed. Herculase II Fusion Enzyme was used for PCR (600677 Agilent Technologies). The master mix shown in Table 3 was used according to the manufacturer’s protocol. Every PCR protocol included 30 cycles.

**Table 1:** PCR protocol for standard PCR program

Initial denaturation	98 °C	2 min
Denaturation	95 °C	20 s
Annealing	53–58 °C target dependent	20 s
Elongation	72 °C	56–69 s target dependent
End elongation	72 °C	3 min

**Table 2:** Primers for PCR and DMSO concentrations for primer pair used

Forward primer [5′–3′]	Reverse primer [5′–3′]	DMSO for subunit primer pair
CAGACCCACCTCCCAACC	GTGCTCCAAGTGCTCCCAG	8% for α1
GAAGTCCGACCAAGAGAGC	GCCCCAAGATCCACACAC	8% for β1
CATTTGCCTCCACCTGATCC	CCAAGCAATCCAAGAAGCAG	5% for δ
GCCTGGATTTTTCTGCAAGGC	GAGTTCTTGCAGCTCGGTGAC	5% for ε

**Table 3:** Master mix of performed PCR

Compounds	Volume (μL)
5× Herculase II buffer	10
dNTPS, each 25 mM	0.5
Template	1
Primer, each 10 μM	1.25
Herculase II	1
DMSO	5% = 2.5 μL; 8% = 4 μL
Nuclease-free water	Fill up to 50 μL

Gel electrophoresis was performed after PCR to separate PCR products in E-Gel EX 1% agarose gels (G401001 Invitrogen) in the E-Gel Power Snap by Invitrogen. Then, target fragments were cut out from the gel under UV light and purified with ReliaPrep DNA clean-up and concentration System by Promega as described before in the section “Cloning strategy.” The correctness of subunit sequences was analyzed with Mix2Seq Kit (Eurofins Genomics). Sequence data was checked with the online tool http://multalin.toulouse.inra.fr.

### ICW with different substances to increase yield of nAChR subunits

In the case of the one target transduction with nAChR, the protein detection was carried out by ICW, OCW, and Western blot. To determine a possible increase in protein levels, four chemical chaperones were tested. Therefore, suitable substances should be small to enter the nAChR binding pocket and polar to bind aromatic amino acids within the binding pocket (Sine 2012). Candidates with these properties besides nicotine are cytisine, choline, and TMA. Further, four substances were used which change physiological pathways. Forskolin induces cAMP and as a result supports a better posttranslational modification by phosphorylation (Green et al. 1991b). Lactacystin and MG-132 are specific ERAD inhibitors at the 26S proteasome (Christianson and Green 2004). SAHA is a histone deacetylase inhibitor (Hockly et al. 2003) and was shown to also induce CN and BiP which increased GABA receptor expression (Di et al. 2013). Table 4 lists these substances and their used concentration and incubation time. Analysis of the effect of every substance was performed with ICW as described before in the section “In-cell Western and on-cell Western.” The best results were additionally tested in OCW to find out whether the internalization rate can be increased. The concentration of compounds was chosen to be non-cytotoxic after a separate determination of cell viability by XTT assay to avoid an additional reduction in the viability of the transduced cells. Furthermore, cells were tested for the same yield-increasing effects with decreased culture temperature at 34 °C for 7 days and 31 °C for 48 h.

**Table 4:** Substances used for increasing protein levels of nAChR

Substance	Concentration	Incubation time
Nicotine	30 μM	24 h
TMA	1 mM	24 h
Choline	10,000 μM	24 h
Cytisine	1 nM	24 h
Lactacystin	2.5 μM	4 h
MG-132	0.5 μM	4 h
Forskolin	20 μM	4 h
SAHA	2.5 μM	24 h

### Statistics

Data analysis was performed by exporting data of Odyssey CLx for ICW and OCW results and exported data of TECAN infinite M200 PRO for XTT results. GraphPad Prism 9.5.1 (733) software was used for graphic presentation and a two-sample *t*-test with dependent samples was applied. *p* values were calculated and values less than 0.05 were considered statistically significant.

## Results

### Viability of transduced cell lines

All generated stable CHO cell lines were tested for their viability. The cell lines were differentiated according to their transduction method. On the one hand one-target transduction (1-TD) and on the other hand two-target transduction (2-TD) were tested. 2-TD were further divided into direct transduction (both targets together) and two consecutive one-target transductions (in steps). The reason for these different methods was to find the balance between protein yield and cell viability since the cell host must handle a complex protein of four different subunits in the case of nAChR and/or the influence of transgenic chaperones.

**Fig. 2:**
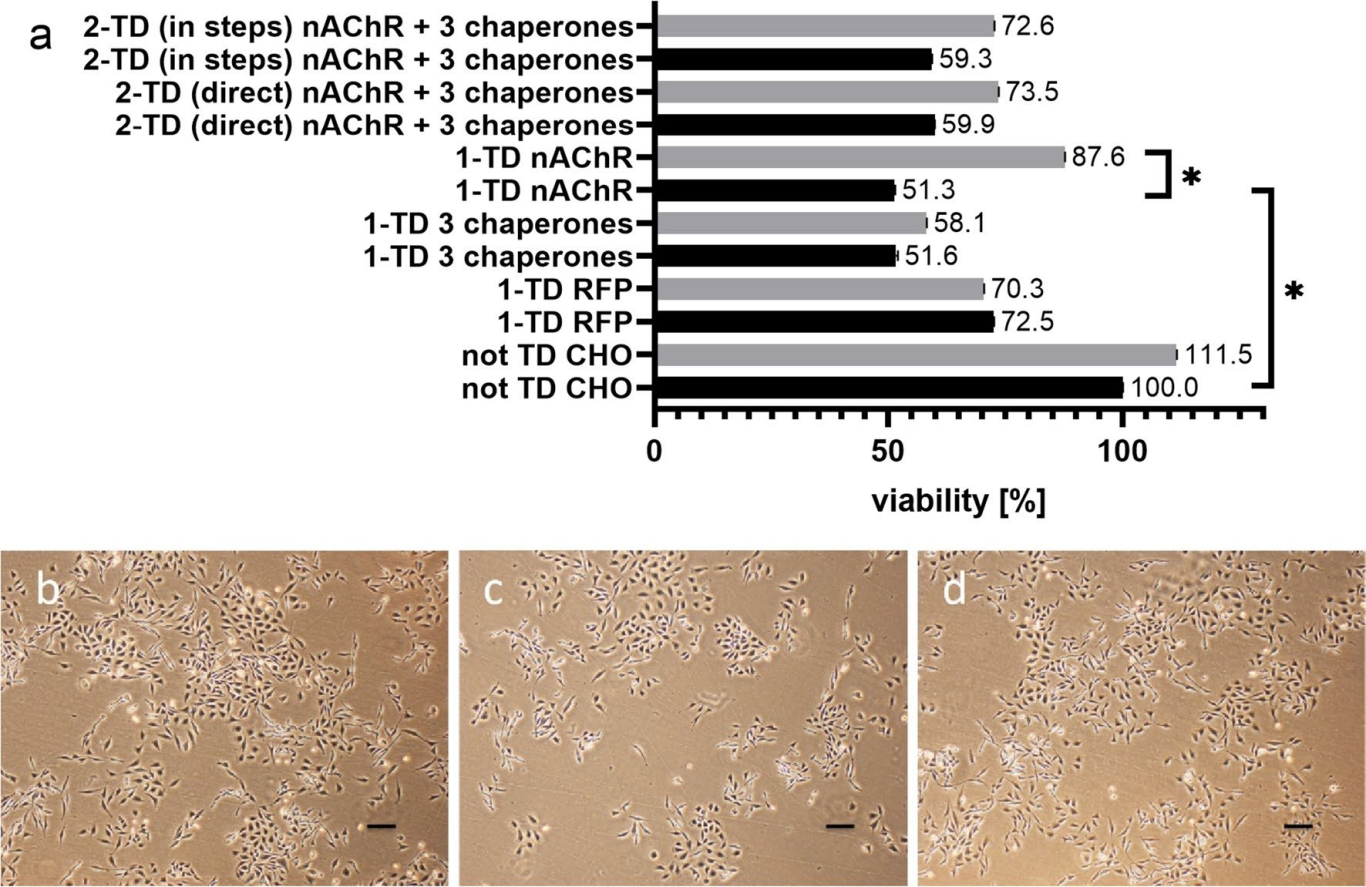
**a** Cell viability of transduced cell lines. Growth conditions with 9% FCS are shown in black and 16.5% FCS in gray. For comparison, untransduced (not TD CHO) cells were grown under the same conditions, and the viability of medium with 9% FCS was set to 100%. Three 1-TD cell lines with an empty backbone (RFP), nAChR, or 3 chaperones showed decreased viability. The viability of the 1-TD cell line with nAChR in comparison to the untransduced CHO cell line decreased significantly. The increased FCS concentration led to an increase in viability, especially in the case of 1-TD with nAChR which was significantly increased in comparison to the 1-TD nAChR CHO cell line grown with 9% FCS. Data are from three biological experiments each with eight technical replicates; the asterisk means significance with a *p*-value below 0.05. Light microscopy images of cell incubation on the same day: **b** untransduced CHO cells under incubating conditions with 9% FCS. **c** 1-TD nAChR cells under incubating conditions with 9% FCS in medium and **d** 1-TD nAChR cells under incubating conditions with 16.5% FCS in medium. Scale bars are 100 μm

As shown in Fig. 2a, all 1-TD cell lines showed decreased viability compared to untransduced cells. Already the transduction with the empty backbone virus vector (RFP) decreased the cell viability to 70% (Fig. 2a, black bars). In the case of 1-TD with nAChR or chaperones, only about 50% viability was detected (Fig. 2a, black bars). Viability of 2-TD procedures showed about a 40% reduction of viability (Fig. 2a, black bars).

In consideration of a healthy cellular model system useful for drug screenings, it was necessary to increase cellular welfare. Translation and posttranslational modifications tie up a lot of cellular capacities in terms of host chaperones and physiological pathways. Thus, the nAChR expression could starve its host, and as compensation, the FCS concentration in the medium was increased from 9 to 16.5%. Figure 2a shows that in the case of the 1-TD nAChR, the viability increased significantly close to 88% (grey bar). The 2-TD direct and in steps procedure showed no difference between each other, but viability was increased to about 73%. Increased FCS concentration had no significant effect for 1-TD for RFP or 3 chaperones (Fig. 2a, gray bars). Figure 2b shows untransduced CHO cells incubated with 9% FCS in the medium, 2c 1-TD nAChR cells under incubation with 9% FCS in the medium, and 2d 1-TD nAChR cells incubated with 16.5% FCS in the medium 2 days after plating the same cell numbers.

### Protein detection

**Fig. 3:**
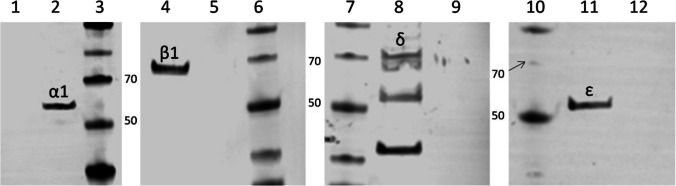
Western blot of transduced (nAChR) and untransduced CHO cell lanes. The detection of subunits was performed on separate blots. Lanes 1, 5, 9, and 12 show CHO cells without transduction for α1 (1), β1 (5), δ (9), and ε (12). Lanes 2, 4, 8, and 11 show CHO cells with nAChR after transduction (1-TD) for α1 (2), β1 (4), δ (8), and ε (11). Lanes 3, 6, 7, and 10 show the marker, 50- and 70-kDa bands are indicated

As evidence of nAChR presence in the established cellular model, the Western blot technique was used. The transduced cell line expressing nAChR (1-TD nAChR) shows the presence of α1, β1, δ, and ε subunits (Fig. 3). As a negative control, untransduced CHO cells were used. CHO cells from 2-TD, transduced with both nAChR and chaperones, did not show the expression of any nAChR subunit (data not shown).

**Fig. 4:**
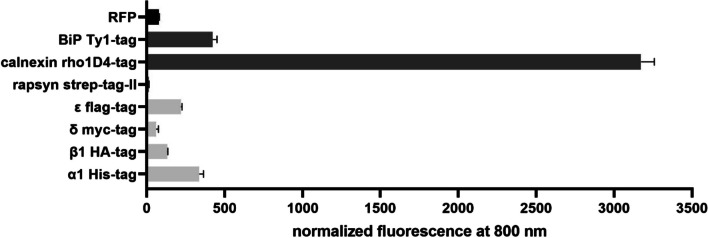
In-cell Western of CHO cell lines transduced with one-target approach (1-TD). Target proteins were detected by their specific tag and fluorescence signals were normalized to 30,000 cells. The black bar is the empty backbone vector with the reporter RFP from 1-TD RFP. Dark gray bars are the three chaperones with their tags strep-tag-II, rho1D4, and Ty1 from 1-TD 3 chaperones. Light gray bars are the subunits of nAChR with their tags His, HA, myc, and flag from 1-TD nAChR. Data are from three biological experiments with nine technical replicates each.

The Western blot method is time-consuming and large amounts of cells are required. Therefore, further protein detections were performed afterward using the ICW method. Additionally, ICW allows native protein detection in whole cells in comparison to their denatured state in Western blot. Thus, since the above-used antibodies were not suitable for detecting the native state, antibodies against the tags were used for ICW instead. Moreover, using antibodies against the tags made it possible to distinguish transgenic chaperone expression from the intrinsic one. By ICW, proteins were separately detected by their protein tags, and fluorescence signals were normalized to cell counts (Fig. 4). RFP levels from the 1-TD approach using the empty backbone vector should serve as a comparison for expression levels gained from unmodified and thus smaller target vectors. Expression of all three chaperones could be verified in CHO transduced with chaperones as 1-TD, while rapsyn showed only a small expression and calnexin was highly expressed. The 1-TD approach transduced with nAChR showed the presence of all four receptor subunits around the same levels with δ as the lowest and α1 as the highest expression. In the case of the 2-TD, i.e., both target plasmids for nAChR and chaperones in one CHO cell host, the signals of nAChR tags were below the detection limit and those of chaperones were decreased (data not shown). The ICW analysis was performed several times over months and the results were comparable (data not shown), indicating that protein detection was possible over long periods; and thus, the cell line could be considered as stable.

Since nAChR protein expression from the 2-TD approach could not be detected by Western blot or ICW, genome integration was verified by genomic PCR as well as sequencing (Figures S1–S5 in Supplementary Information). Indeed, all four nAChR subunits are present in the corresponding genomic PCR product and the sequences were correct.

### Functional analysis

**Fig. 5:**
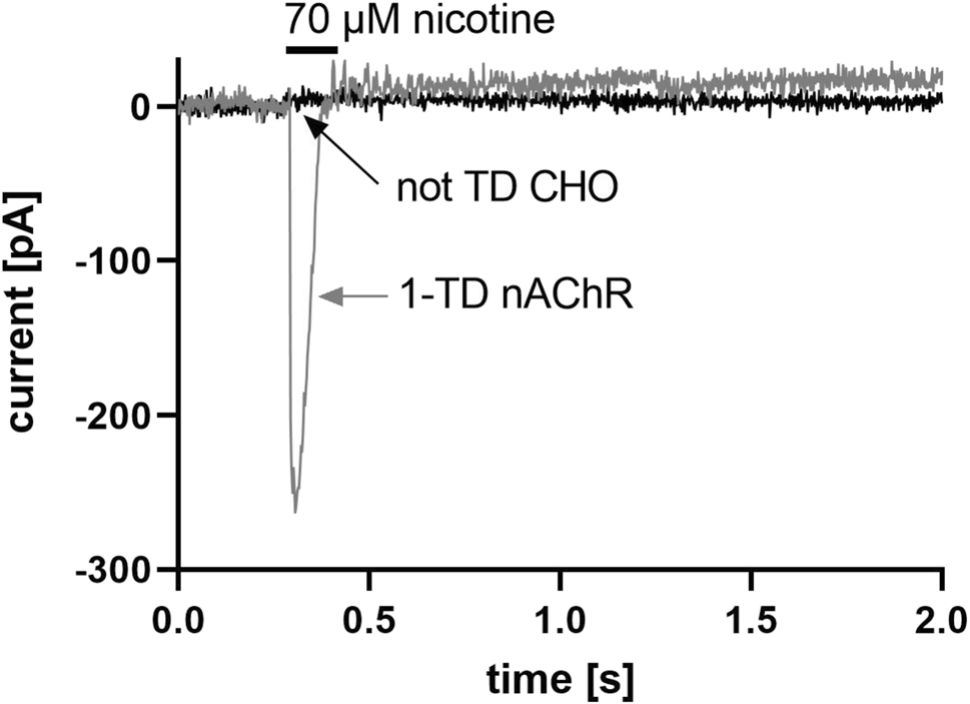
Representative whole-cell currents in 1-TD nAChR and not TD CHO cells. The black current corresponds to untransduced CHO (not TD CHO) and gray current to nAChR (1-TD nAChR) cells. The traces show the current response to 15 μL of 70 μM nicotine applied at a rate of 114 μL/s. The black horizontal bar indicates the exposure time. Currents were elicited at a holding potential of −70 mV

For functional analysis of the nAChR, whole-cell patch-clamp recordings were performed. Figure 5 shows representative traces of currents from nicotine application in 1-TD nAChR and the untransduced CHO cell line which served as control. In 1-TD nAChR cells, a current intensity of about – 265 pA and response time of 0.3 s could be detected during nicotine application, whereas untransduced CHO cells showed no response.

### Increase of nAChR protein yields

After providing evidence of the presence of nAChR and chaperones by their tags and an increased viability of cellular host by increased FCS concentration in the growth medium, substances from Table 4 were used to increase the yields of nAChR subunits. Incubation times and concentrations are listed in Table 4 in the section “ICW with different substances to increase yield of nAChR subunits.” Additionally, cells were incubated at 34 °C for 7 days or at 31 °C for 48 h. nAChR detection was performed by ICW and results are shown in Table 5.

**Table 5:** Overview of increased yields of every subunit

Compound	α1 (%)	Signif.	β1 (%)	Signif.	δ (%)	Signif.	ε (%)	Signif.
30 μM nicotine	33	*	22	*	31	*	14	*
1 mM TMA	31	*	20	*	28	*	10	*
10 mM choline	25	*	10	*	27	*	10	*
1 nM cytisine	19	*	3		15		0	
2.5 μM lactacystin	27	*	25	*	22	*	2	
0.5 μM MG-132	29	*	19	*	25	*	4	
20 μM forskolin	25	*	0		5	*	0	
2.5 μM SAHA	22		0		17	*	2	
30 μM nic.+ 0.5 μM MG-132	40	*	30	*	29	*	15	*
30 μM nic.+ 10 mM choline	32	*	13	*	29	*	6	
30 μM nic.+ 20 μM forskolin	26		2		20	*	2	
31 °C for 48 h	66	*	83	*	79	*	72	*
34 °C 7 days	0		0		5		5	

Nicotine, TMA, choline, and cytisine were used as chemical chaperones, and lactacystin, MG-132, forskolin, and SAHA as substances supporting posttranslational modification. Moreover, the combinations of both compounds as well as hypothermic incubation at two temperatures were tested. α1/β1/δ/ε means increased subunit protein levels after treatment in percentage compared to the corresponding subunit level in the absence of any treatments (%). *Signif.*, significance of the increased effect for the corresponding subunit

*Significance with *p*-value below 0.05

Substances, temperatures, and their effects are sorted in Table 5 according to (i) chemical chaperones, (ii) substances supporting posttranslational modification, (iii) best combination of both, and (iv) incubating temperature. Within each group, the yield increases are in decreasing order, and yields are shown in comparison to the corresponding subunit without the substance treatment whose signal was set to 0%.

Results for (i) chemical chaperones show that nicotine was the best substance out of this group. TMA and choline had similar but slightly less efficient effects than nicotine on the subunits. The effect of cytisine was only significant for the α1 subunit. Using class (ii) substances supporting posttranslational modifications, the ERAD inhibitors lactacystin and MG-132 led to increased subunit yields. Forskolin had significantly increased effects for two subunits and SAHA only for the δ subunit. In the case of (iii), compound combination, nicotine, and MG-132/choline/forskolin increased subunits differently. However, the protein level increase by the combinations did not significantly exceed the average of the sole application of nicotine.

**Fig. 6:**
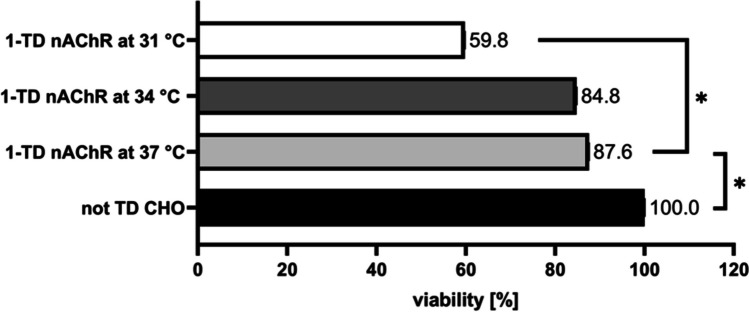
Cell viability of 1-TD nAChR cell line under hypothermic conditions. The black bar indicates untransduced (not TD CHO) cells which were grown at 37 °C and 9% FCS and their viability was set to 100%. 1-TD with nAChR at 37 °C and 16.5% FCS (light gray) showed a viability of about 88%. Viability of 1-TD with nAChR at 34 °C for 7 days and 16.5% FCS (dark gray) is minimally decreased. Viability of 1-TD with nAChR at 31 °C for 48 h and 16.5% FCS (white) is significantly decreased in comparison with 1-TD nAChR at 37 °C. Data are from three biological experiments each with eight technical replicates, * means significance with *p*-value below 0.05.

In the case of (iv), hypothermia there was a huge increase of all subunits at 31 °C incubation for 48 h, which even exceeded all other tested conditions. In comparison, incubation at 34 °C for 7 days in total with sub-incubation on the third day did not result in increased yields (Table 5). However, cell incubation at sub-optimal temperature will always show decreased viability, and to measure the extent of this effect, viability at both hypothermic conditions was tested. As shown in Figure 6, the reduction of incubation temperature to 34 °C for 7 days in total showed a not significant decrease. In contrast, the reduction to 31 °C for 48 h significantly reduced cell viability to about two-thirds of those at 37 °C. Thus, combining the results from protein yields and cell viability at hypothermia, at 34 °C, no increase in protein yield but unaffected viability was shown, while at 31 °C, the yields were massively increased but the viability was substantially decreased at the same time. Overall, the best effect regarding nAChR yield and cell viability was obtained by the growth medium added with 30 μM nicotine at otherwise standard culture conditions.

**Fig. 7:**
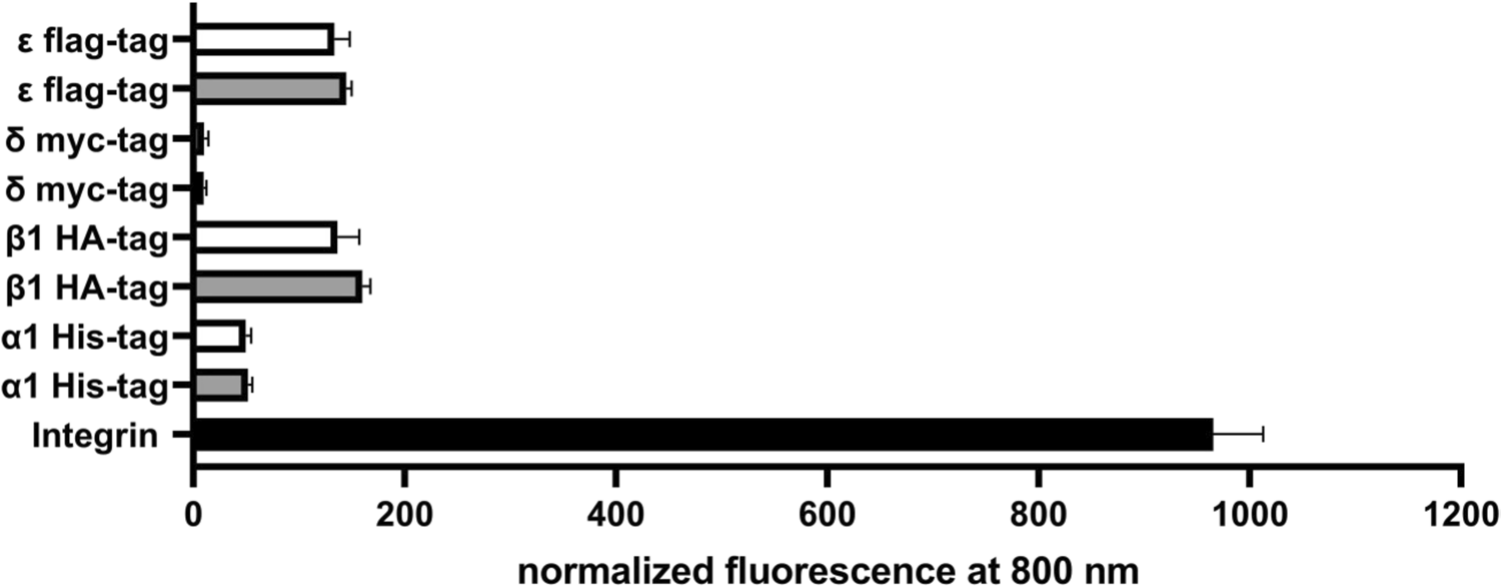
On-cell Western of CHO cells transduced as one target (1-TD nAChR). Target proteins were detected by their specific tag in native conditions and fluorescence signals were normalized to 30,000 cells. The housekeeper protein integrin (black bar) was used as a reference. Gray bars are the subunits of nAChR with their tags His, -HA, -myc, and -flag from 1-TD without incubation of nicotine. White bars are the subunits of nAChR with their tags His, -HA, -myc, and -flag from 1-TD with incubation of 30 μM nicotine for 24 h. Signals are from three biological experiments with six technical replicates

In OCW, it was tested whether this increased yield by adding 30 μM nicotine is also reflected by increased internalization of the nAChR into the plasma membrane (Fig. 7). Fluorescence signals were normalized to cell counts as in ICW. The signals in OCW showed no significant difference between the presence or absence of nicotine for all subunits tested. To compare the level of internalization of the subunits, the signal of the housekeeper integrin was detected and shown as a reference.

## Discussion

This study describes the stable expression of a functional human muscle-type nAChR in CHO cells for the first time. For the expression of such a complex transmembrane protein, several improvements were tested. nAChR expression initially decreased the cell viability by 50%. The cellular host might be starved in order to correctly assemble the receptor since several chaperones are involved in this process like BiP which needs ATP (Marinko et al. 2019); and thus, the medium with 9% FCS might not be substantial enough. This statement is supported as the increase of FCS concentration to 16.5% increased the viability to 88% (Fig. 2). Western blot analysis was performed and all four nAChR subunits could effectively be detected. Since nAChR is a transmembrane protein, the solubilization and affinity purification of nAChR are inefficient and elaborate (Maldonado-Hernández et al. 2020). Moreover, large amounts of the cells are needed and only the denatured form of proteins can be detected. Thus, ICW and OCW were used as additional and comparable methods to detect the complex transmembrane nAChR, which only required a few thousand cells, and the native state could be detected. Using these methods, we showed that nAChR could be detected intracellularly but maybe even more importantly, on the cell surface. Moreover, we could provide functional evidence for the generated cell line. For the first time, a functional analysis of human muscle-type nAChR (Figure 5) was performed; and thus, a comparison of the functional performance with other studies is difficult. The current intensities of systems naturally expressing nAChR seem to be higher (Maconochie and Knight 1992) than the one measured in this study and other cellular models. However, the current of our study is comparable in terms of response time and current intensity to mammalian cell models with recombinant α7 nAChR (Williams et al. 2005; Gu et al. 2016; Scheffel et al. 2018b).

Moreover, the hypothesis to increase receptor yields by (i) biological or (ii) chemical chaperones, (iii) substances supporting posttranslational modification, and (iv) hypothermia was tested. Due to the complex assembly and posttranslational modifications of muscle-type nAChR, it was hypothesized firstly that additional biological chaperones might be needed. So far, other studies with nAChR already showed inefficient yields without chaperones (Merlie and Lindstrom 1983; Green and Wanamaker 1998; Wanamaker et al. 2003). It was a challenge to generate a viable recombinant cell line of nAChR and three biological chaperones since the cellular host has to accomplish the biogenesis of the receptor built up of four subunits and the influence of transgene chaperones as well as the cellular metabolism needs to handle overexpression of seven different proteins. Therefore, 2-TD direct and in steps were performed to evaluate which was more suitable for cell viability and receptor yield. Figure 2 shows that the viability of both approaches resulted in only about 60% viability, which could be increased to about 73% using higher FCS concentration. However, this is still a lot less than the 88% of 1-TD nAChR approach. Moreover, nAChR protein could not be detected in 2-TD cells and the levels of chaperones were drastically reduced even if genome integration and sequence correctness of all four nAChR subunits could be shown. This is in line with the literature as transient, not human muscle-type nAChR co-expressed with CN, BiP, and ERp57 also resulted in missing or decreased receptor expression (Wanamaker and Green 2007). Since both viability and receptor yields were negatively affected in our study, biological chaperones were not further investigated. However, other chaperones not known so far may act more specifically on muscle-type nAChR and their overexpression could have a more positive effect than the chaperones tested in our study.

Secondly, the yield-increasing effects of chemical chaperones were investigated (Table 5). Chemical chaperones were used to support the receptor assembly and may affect the nAChR more specifically than biological chaperones. Chemical chaperones may not have the same effect on all subunits but only operate on the subunit or dimer to which they bind. Thus, every chemical chaperone has different efficiencies to increase the yield of each subunit. We could show that nicotine had the best results. This is in line with data that different neuronal nAChR types were also induced with nicotine (Flores et al. 1992; Sallette et al. 2004; Xiao and Kellar 2004). At the same time, nicotine showed no decreased cellular viability. However, the increase of protein levels by nicotine did not result in a higher internalization of the receptor into the plasma membrane (Fig. 7).

Thirdly, substances supporting posttranslational modification were tested for their yield-increasing effects. Green et al. (1991a) and Green et al. (1991b) used forskolin, which leads to cAMP stimulation, which phosphorylated subunits, to increase Torpedo nAChR in mouse fibroblasts; and thus, the δ subunit yield was minimally increased. Our data showed the same effect (Table 5). Moreover, the γ subunit of Torpedo nAChR was also increased, which is crucial for trimer building (Green et al. 1991a). In human muscle nAChR, the ε subunit replaces the γ subunit. Since forskolin did not increase the ε subunit in our study, the assembly of the receptor might be different from that currently expected. However, the tested substances of this class were less effective than chemical chaperones.

Lastly, hypothermia was tested for increased yields. Growth at 34 °C for 7 days did not improve receptor yields but cell viability was not affected. Incubation at 31 °C for 48 h indeed highly increased the yields of all subunits, which even exceeded the nicotine results. However, cell viability was significantly decreased accordingly. These conditions tested are not favorable but maybe shorter hypothermia periods may increase nAChR expression without affecting cell viability drastically.

So far, nicotine seems to be the best condition tested and favorable in terms of increased nAChR subunit yields as well as not affecting cell viability. However, nicotine did not increase nAChR levels at the cell surface and we cannot provide information on whether the increased yields also reflect correctly folded and/or functional proteins. Further studies should evaluate this deficiency, and this might improve our cellular model system even more.

To conclude, this study shows a functional cellular model system for stable recombinant human nAChR expression. The established transgene CHO cell line is capable of nAChR protein expression with only minimally decreased cell viability. A variety of conditions were tested to increase the protein yield, out of which nicotine showed the best results. The presented transgene CHO cells provide a useful tool in drug screening studies and various other research areas.

### Supplementary Information

ESM 1 (DOCX 791 kb)

## Abbreviations

BiP: Binding immunoglobulin protein
CHO: Chinese hamster ovary
CN: Calnexin
EDTA: Ethylenediaminetetraacetic
ER: Endoplasmic reticulum
ERAD: Endoplasmic reticulum–associated degradation
FCS: Fetal calf serum
ICW: In-cell Western
nAChR: Nicotinic acetylcholine receptor
OCW: On-cell Western
rapsyn: Receptor-associated protein at synapse
SAHA: Suberoylanilide hydroxamic acid
TD: Target transduction
TMA: Tetramethylammonium
