# Supplementary material for: Recombinant cellular model system for human muscle-type nicotinic acetylcholine receptor α12β1δε
Source: Cell Stress Chaperones. 2023 Nov 25;28(6):1013–25. doi: 10.1007/s12192-023-01395-0 (PMC10746606; doi:10.1007/s12192-023-01395-0)
Supplement: Supplementary file 1 — (DOCX 791 kb) [file 12192_2023_1395_MOESM1_ESM.docx]

**Title**

Recombinant cellular model system for human muscle-type nicotinic acetylcholine receptor α1_2_β1δε

**Journal name**

Cell Stress and Chaperones

**Authors**

Sabrina Brockmöller*, Thomas Seeger, Franz Worek, Simone Rothmiller

**Affiliations**

Bundeswehr Institute of Pharmacology and Toxicology, Munich, Germany

*** Corresponding author**

[Sabrina.Brockmoeller@yahoo.de](about:blank), +49 (0) 89-992692-2927

**Supplementary Information**


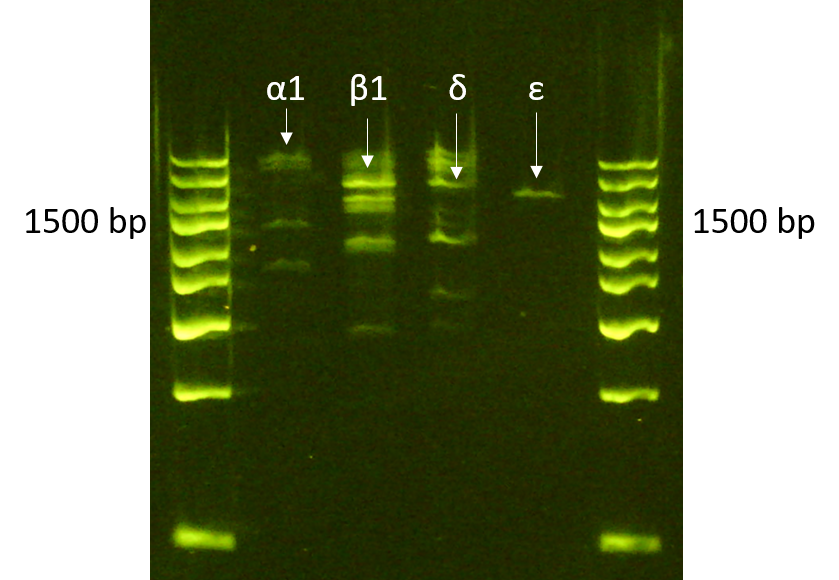


Figure S1: Gel electrophoresis of genomic PCR performance of 2-TD (direct) nAChR CHO cell line transduction. The marker was added on the left- and right-hand side and 1500 bp is indicated. Corresponding subunit bands were explicitly shown by arrows. All subunits of nAChR were present.


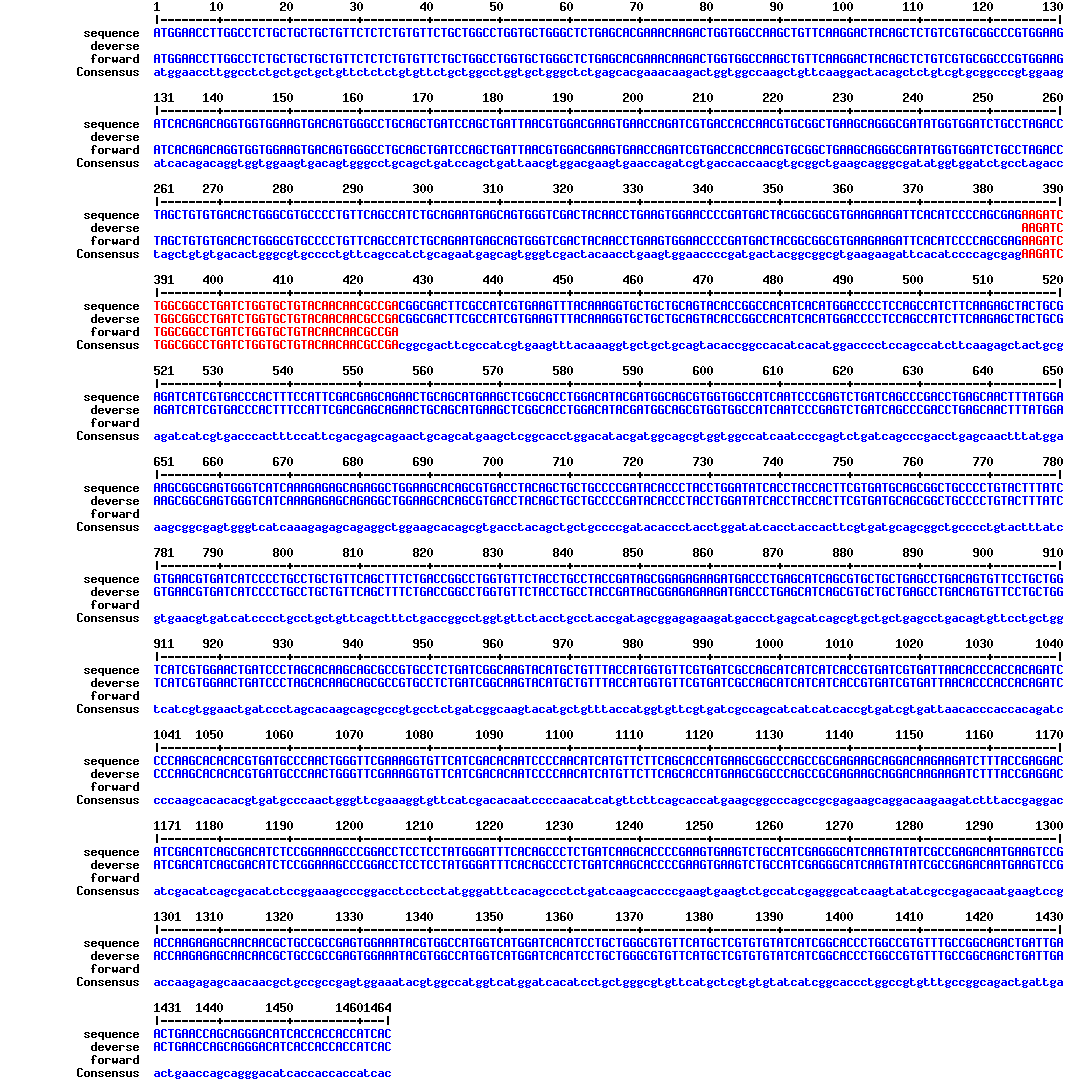


Figure S2: Sequence analysis data from α1 subunit with His-tag created with online tool multalin.toulouse.inra.fr. Sequence represented the template, deverse represented the analysed sequence of reverse primer, forward represented the analysed sequence of forward primer, and consensus represented the correctness of both from deverse and forward.


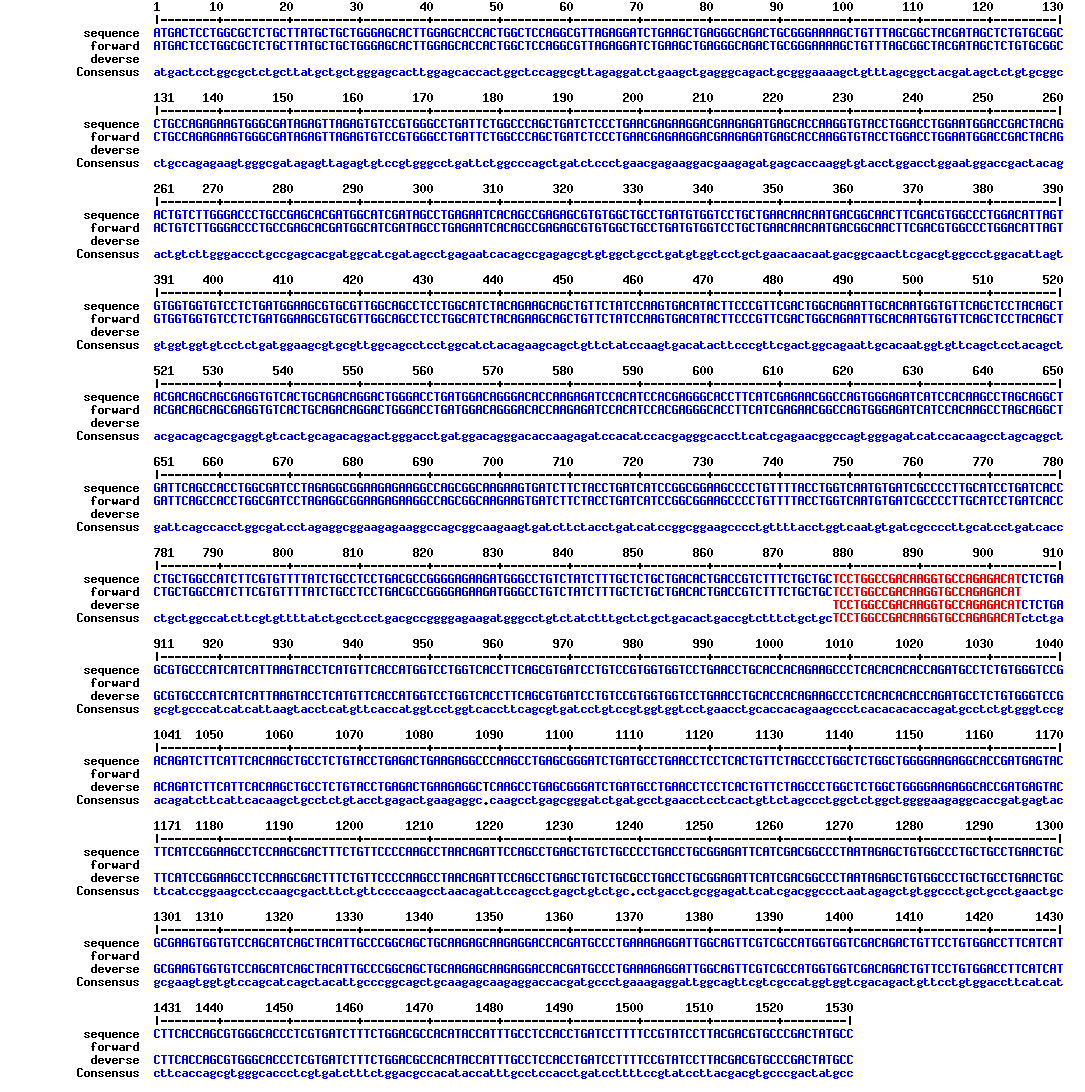


Figure S3: Sequence analysis data from β1 subunit with HA-tag created with online tool multalin.toulouse.inra.fr. Sequence represented the template, deverse represented the analysed sequence of reverse primer, forward represented the analysed sequence of forward primer, and consensus represented the correctness of both from deverse and forward.


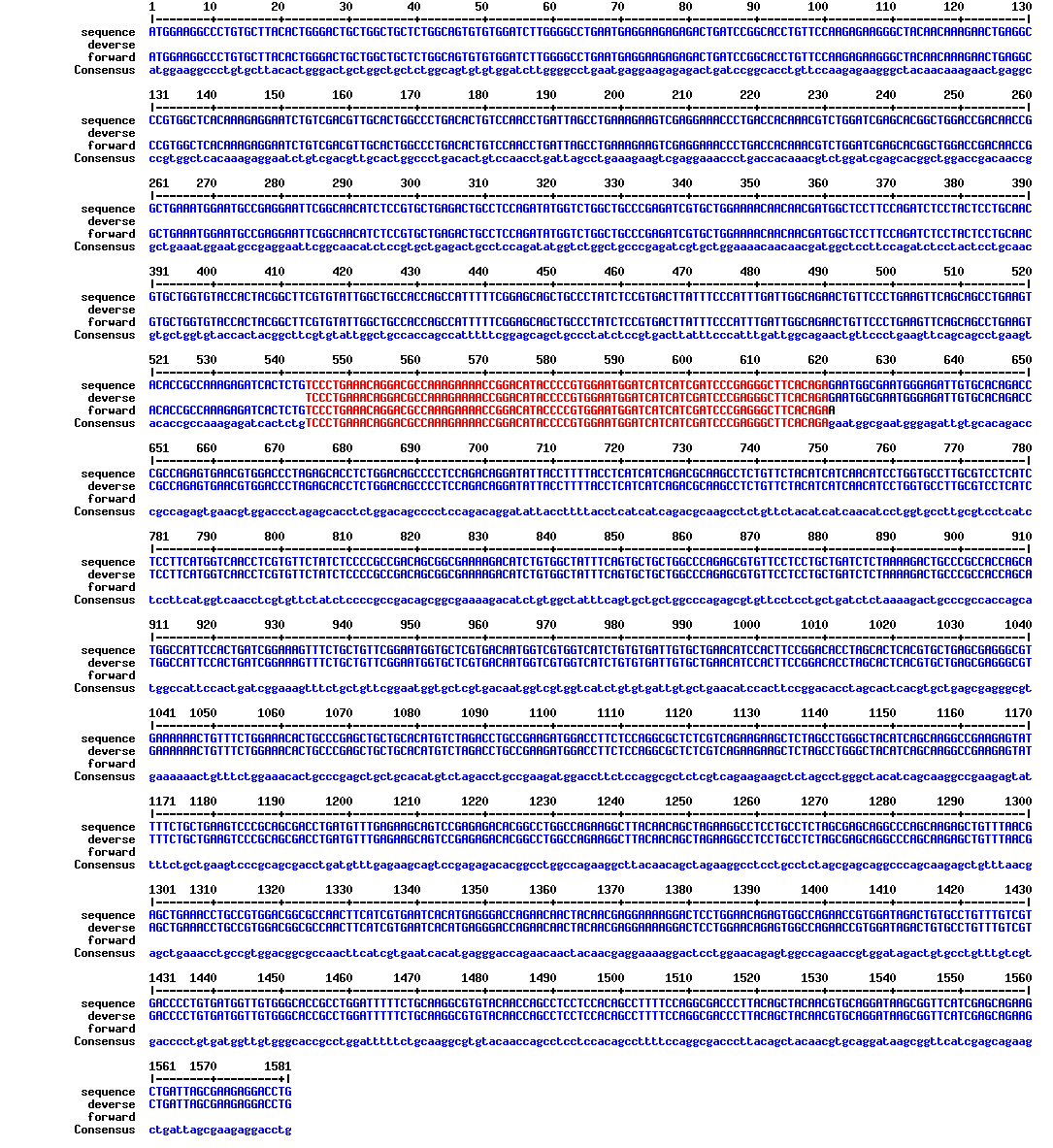


Figure S4: Sequence analysis data from δ subunit with myc-tag created with online tool multalin.toulouse.inra.fr. Sequence represented the template, deverse represented the analysed sequence of reverse primer, forward represented the analysed sequence of forward primer, and consensus represented the correctness of both from deverse and forward.


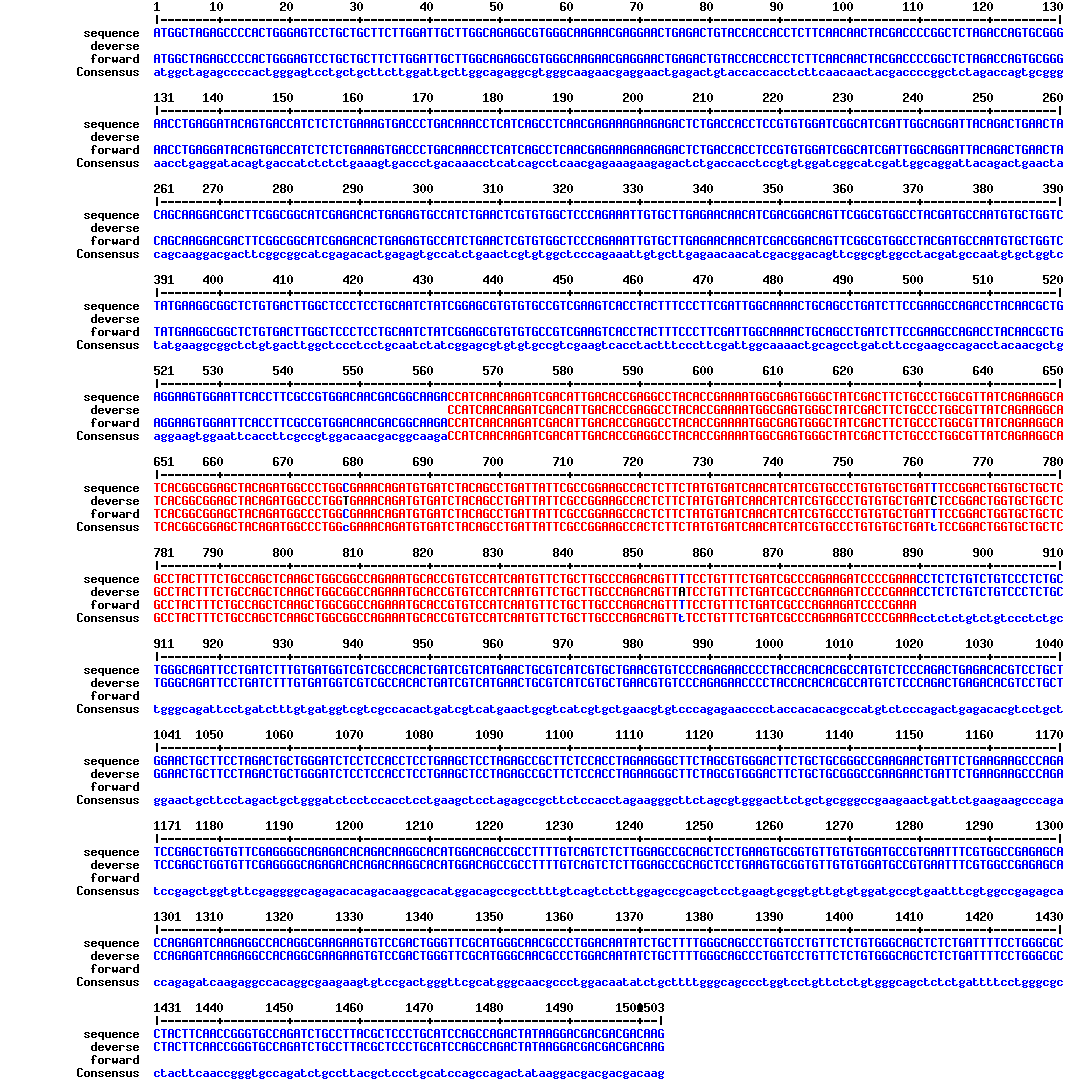


Figure S5: Sequence analysis data from ε subunit with flag-tag created with online tool multalin.toulouse.inra.fr. Sequence represented the template, deverse represented the analysed sequence of reverse primer, forward represented the analysed sequence of forward primer, and consensus represented the correctness of both from deverse and forward.
